# Acute respiratory distress syndrome in a patient with tuberculosis

**DOI:** 10.2144/fsoa-2019-0158

**Published:** 2020-07-30

**Authors:** Rabih Hallit, Souheil Hallit

**Affiliations:** 1 Faculty of Medicine & Medical Sciences, Holy Spirit University of Kaslik (USEK), Jounieh, Lebanon; 2 Department of Infectious Diseases, Bellevue Medical Center, Mansourieh, Lebanon; 3 Department of Infectious Diseases, Notre Dame des Secours University Hospital, Byblos, Lebanon; 4 INSPECT-LB: Institut National de Sante Publique, Epidemiologie Clinique et Toxicologie, Beirut, Lebanon

**Keywords:** acute respiratory distress syndrome, mortality, tuberculosis

## Abstract

Tuberculosis (TB) continues to be a major cause of death worldwide and can have varying manifestations. Acute respiratory distress syndrome (ARDS) is a rare complication of the clinical course of TB but carries a high mortality rate. We present a case of a diabetic African-American patient, with acute respiratory failure, rapidly progressing to ARDS secondary to TB, which had a fatal outcome. Clinicians should keep a high suspicion index for TB in the setting of ARDS since the use of empiric anti-TB treatment could potentially reduce mortality in these patients.

## Lay abstract

Tuberculosis (TB) is a common infection that can affect the lungs. Acute respiratory distress syndrome rarely occurs with TB but carries a high mortality. Early treatment is important because it can reduce mortality. We present a case of TB complicated by acute respiratory distress syndrome, which had an extremely poor outcome.

Tuberculosis (TB) is the most common cause of death due to a single infectious agent worldwide [1]. Although most patients present with a chronic illness of insidious onset, presentation with acute respiratory failure requiring assisted ventilation is well described [2]. Pulmonary TB is a well-recognized cause of this kind of presentation [3]. Some patients may also rarely present with typical features of acute respiratory distress syndrome (ARDS). Mortality from ARDS secondary to TB is around 50% (95% CI: 43–57%) [4].

## Case presentation

We present a case of a 47-year old African-American male, who came to the hospital complaining of diffuse abdominal pain and vomiting for 4 days. His past medical history included diabetes mellitus, chronic pancreatitis and chronic renal insufficiency. When the patient was in the emergency room, he became hypoxic, in severe respiratory distress and was subsequently intubated. No further history could be obtained from the patient; there was no documented history of prior purified protein derivative, recent travel, homelessness, or exposure to animals or tick bites. His old records showed that he is a smoker and has a history of alcohol and illicit drug abuse. Physical exam after intubation showed a blood pressure of 155/98 mm Hg, a temperature of 97.7 °F a pulse of 104 beats/min, a respiratory rate of 28 per min and an oxygen saturation of 100% on 100% of FiO_2_. He was cachectic and ill-looking. He had a poor dentition. Pulmonary auscultation revealed decreased breath sounds with bilateral crackles. Heart exam revealed tachycardia with normal heart sounds and no added murmurs. His abdomen was soft without any evidence of peritoneal signs. Rectal exam revealed hemorrhoids, with a positive stool guaiac. Laboratory tests results are summarized in Table 1.

**Table 1.** Laboratory tests and arterial blood gas results.

White blood cells	17,600/μl
Hemoglobin	7.5 g/dl
Platelets	641,000/μl
Liver enzymes	
– Bilirubin	1.5 mg/dl
– Albumin	2.4 g/dl
– ALP	172 units/l
– SGOT	61 units/l
– SGPT	24 units/l
Amylase	550 units/l
Lipase	9 units/l
Blood glucose	59 mg/dl
Arterial blood gas	
– pH	7.32
– PCO_2_	33 mm Hg
– PaO_2_	78 mm Hg
– Bicarbonate	17 mEq/l
– Saturation	94%
FiO_2_	100%

**Figure 1.**
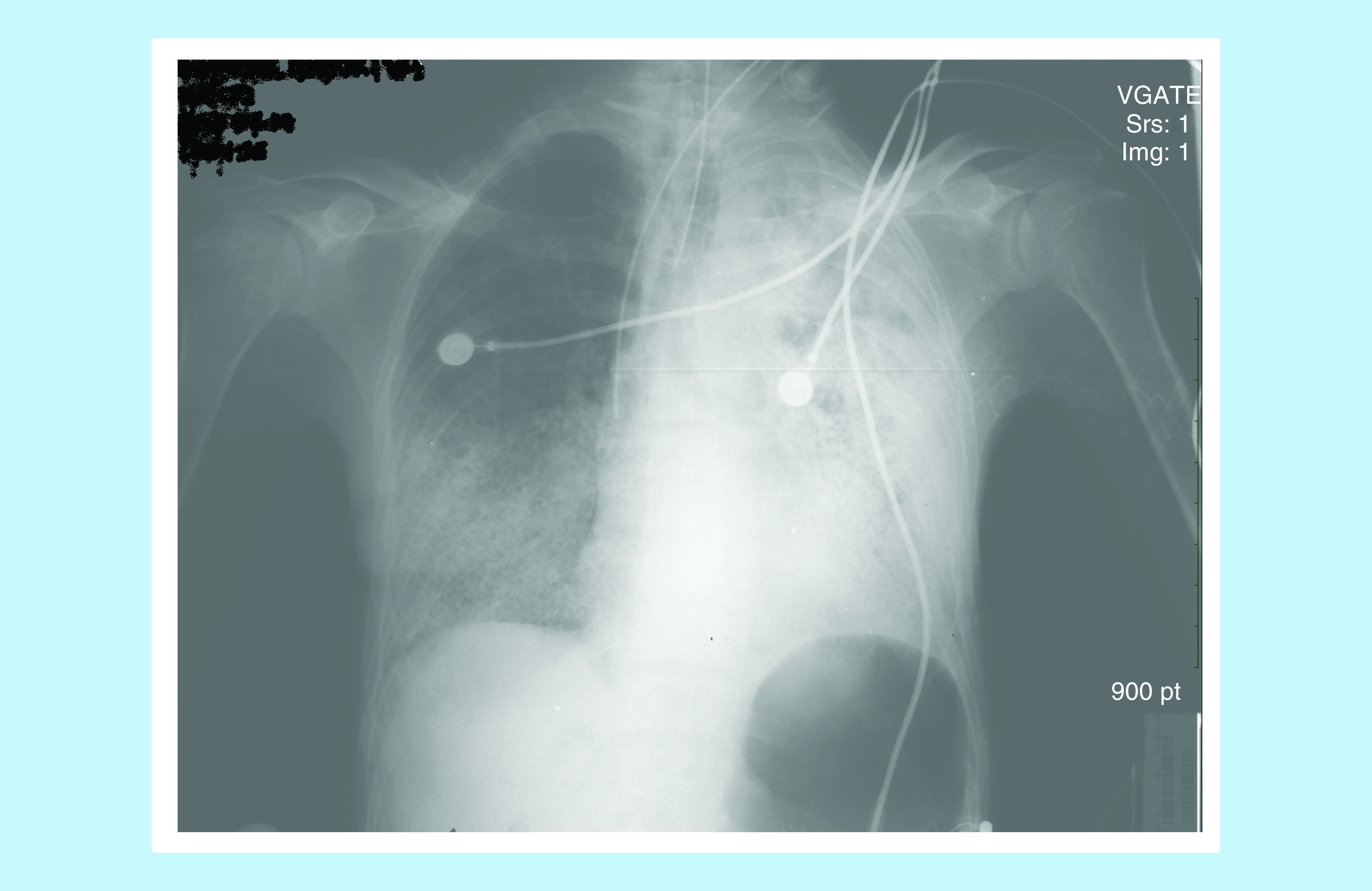
Chest x-ray of the patient with tuberculosis and acute respiratory distress syndrome.

**Figure 2.**
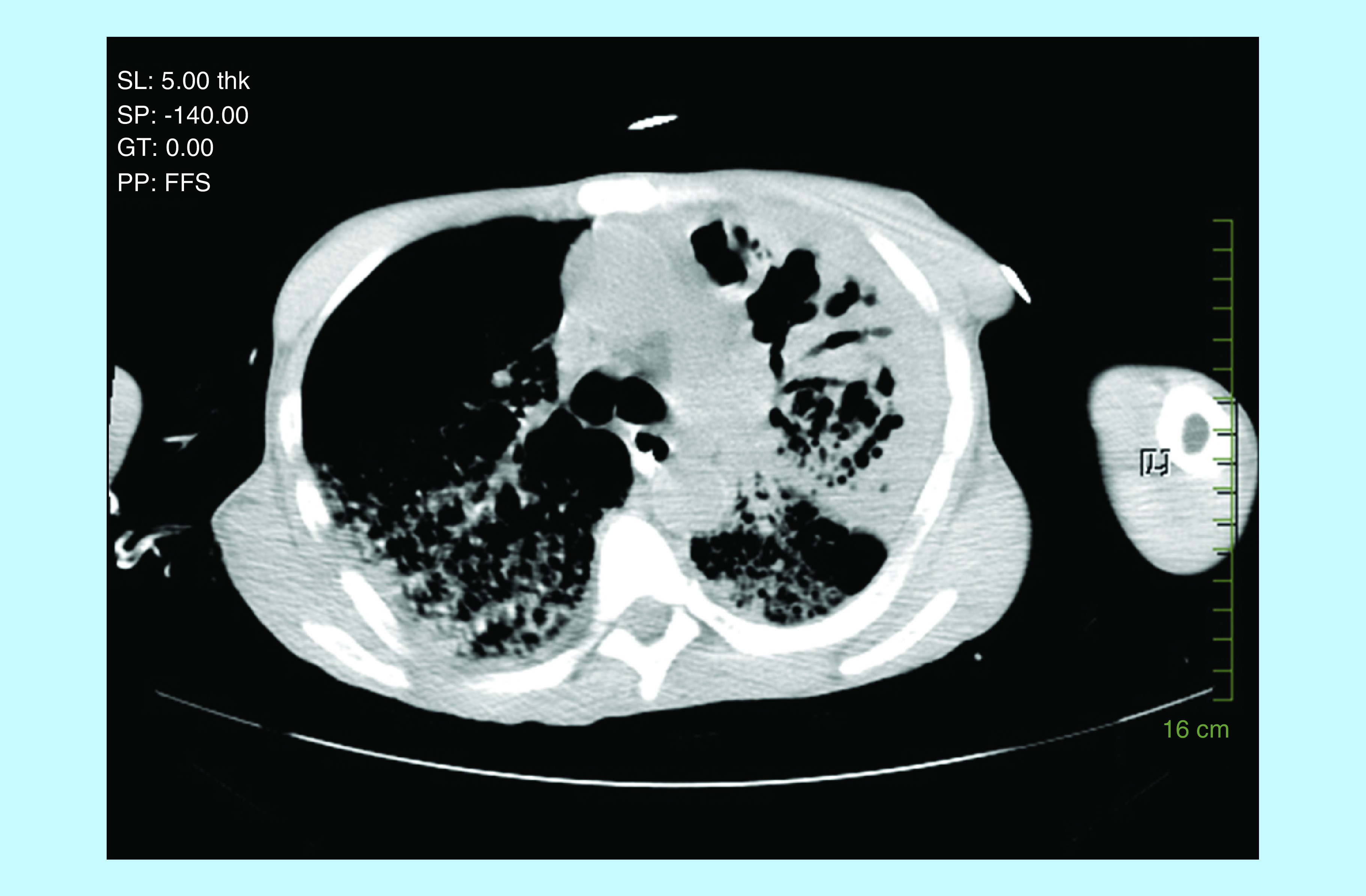
CT scan of the patient with tuberculosis and acute respiratory distress syndrome.

**Figure 3.**
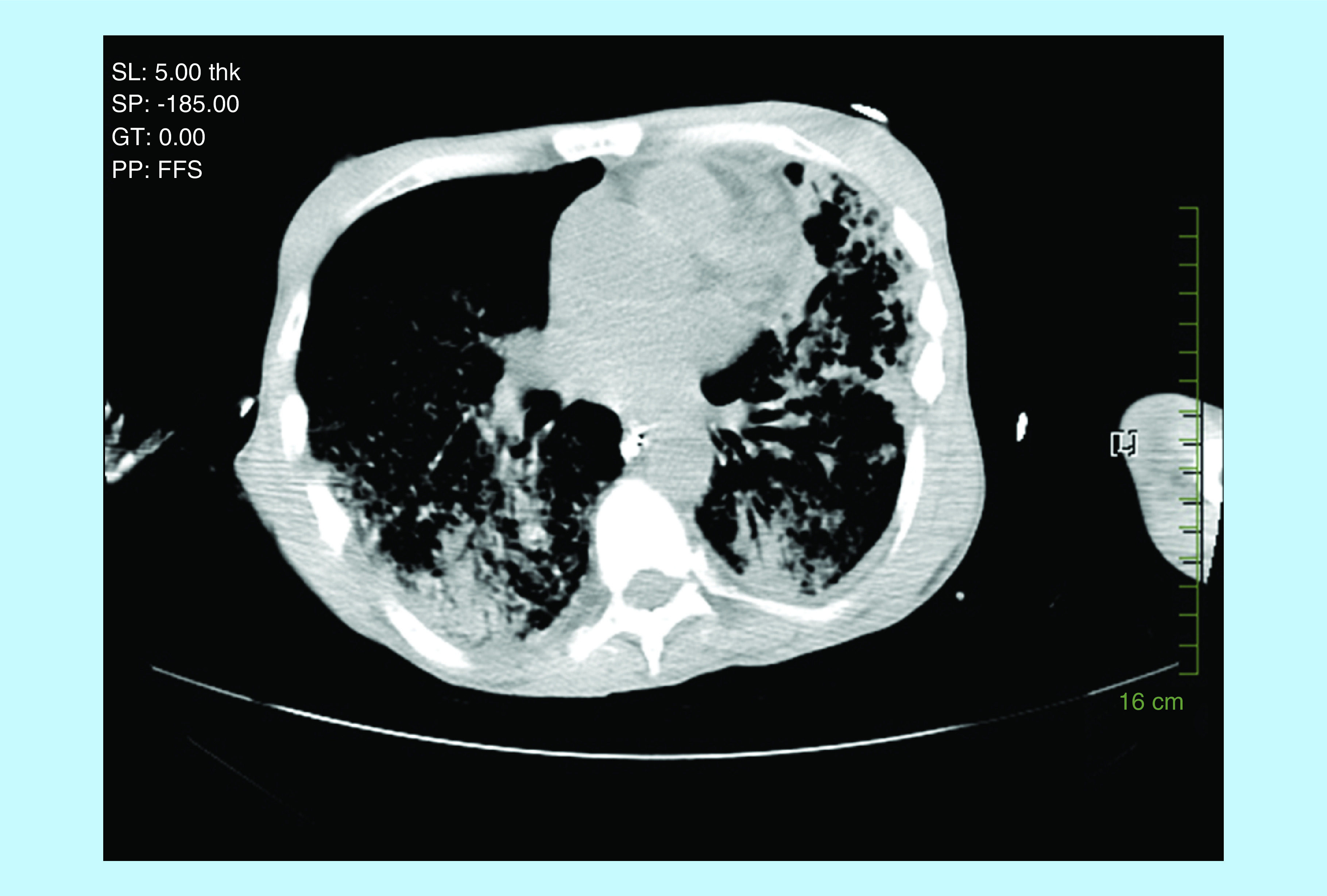
CT scan of the patient with tuberculosis and acute respiratory distress syndrome.

His APACHE score was 24. Chest x-ray showed extensive bilateral air space disease with congestion worse on the left, than the right side (Figure 1). CT scan of the chest confirmed chest x-ray findings, as well as a cavitation in the left upper lobe probably representing necrotizing pneumonia (Figures 2 & 3). CT scan of the abdomen and pelvis revealed a questionable thickening of the transverse and descending colon compatible or suspicious of colitis. The patient was admitted to the intensive care unit and was started on vancomycin, piperacillin–tazobactam and azithromycin. Rapid HIV test came back negative. The patient then coded, but was successfully resuscitated. Acid-fast bacilli (AFB) smear came back 3+ and the patient was started on rifampin, isoniazid, ethambutol and pyrazinamide. The patient’s condition continued to deteriorate; after discussion with his family, a decision not to resuscitate the patient was taken. The patient passed away 48 h after admission. PCR for TB came back positive for mycobacterium TB, with a culture growing a pan-sensitive organism.

## Discussion

TB can have varying clinical manifestations, especially when infection is widely disseminated [2]. Early suspicion and therapy for TB may play a life-saving role in the initial management of patients with ARDS, a rare complication of the clinical course of TB [3,5]. It has been shown that mortality rates in patients with pulmonary TB is 42.9% [2]. Airborne isolation and four-drug treatment regimen consisting of rifampin, isoniazid, pyrazinamide and ethambutol is started, following the collection of sputum for culture. TB is a treatable cause of ARDS. Delayed initiation of antituberculous therapy contributes to morbidity and mortality due to TB infection in developed countries. It has been suggested that in the setting of weight loss, cough and fever, the benefits of antituberculous therapy most likely outweigh the risk of toxicity of these drugs [6–8]. In any case, the clinical suspicion of TB remains the limiting step in the management of TB and prevention of its sequelae, such as ARDS [3,6,7].

Predictors of mortality in TB include APACHE II scores above 18. In intensive care unit patients, the number of organ failures, serum albumin below 20 g/l and a larger number of lobes involved on chest x-ray have been shown to increase mortality [4]. Signs and symptoms to assist in the diagnosis of TB have been well described, but atypical presentations abound. The rapid initiation of antituberculous treatment is essential to the management of patients developing ARDS secondary to TB; the use of steroids in this setting has been described, although its use remains controversial [3,5].

In this patient, the initial suspicion of TB was low, given the chief complaint of abdominal pain, nausea and vomiting, with a benign physical examination of the abdomen. However, the development of acute respiratory failure requiring intubation prompted the collection of a sputum sample to stain for AFB. Although a complete history was unable to be obtained, the patient’s cachectic appearance gave an indication that a chronic process was taking place – a likely onset of symptoms greater than 30 days – and the initiation of antituberculous therapy was initiated within 24 h of admission. Initial treatment based on the patient’s presentation was empiric for bacterial pneumonia with vancomycin, piperacillin–tazobactam and azithromycin. The patient did not have a documented purified protein derivative prior to admission, and the diagnostic work-up revealed extensive airspace disease with interstitial components in both fields, as well as a cavitary lesion in the left upper lobe. This patient received treatment for TB, following a positive smear for AFB, but his prognosis was quite poor and empiric antituberculous therapy would most likely not have altered the outcome.

Despite numerous case studies and anecdotal evidence regarding the development of ARDS in patients with TB, the mortality rate remains high [5–8]. Although a retrospective study suggested that the mortality of TB in ARDS remains comparable with ARDS due to other causes [9], another study in South Korea had opposite results and found that the mortality rate with ARDS caused by miliary TB was 62.3%, which was higher than that in patients with ARDS caused by other diseases [10].

### Teaching message

In all cases, clinicians should keep a high suspicion index for TB in the setting of ARDS, particularly when the patient has risk factors predisposing them to mycobacterial infections. The use of empiric antituberculous treatment could potentially reduce mortality in patients with TB-ARDS, although the time between initiation of therapy and sputum stain for AFB is likely negligible.

## Conclusion & future perspective

Although this rare complication of TB has been well-described, the varying and atypical presentations of patients with TB will continue to make the management of these patients difficult, with concordant high mortality. Further prospective studies with longer follow-ups are necessary to assess the different clinical characteristics in patients with TB complicated by ARDS.

**Executive summary**

**Background**

- Tuberculosis (TB) common cause of death worldwide.
- Acute respiratory distress syndrome (ARDS) a rare complication of TB.

**Case presentation**

- Forty-seven-year old diabetic male.
- Admitted with vomiting and abominal pain.
- Acute respiratory failure and intubation.
- Extensive airway disease on computed tomography chest.
- ARDS.
- Empiric broad spectrum antibiotics.
- Sputum for acid-fast bacilli positive.
- Antituberculous treatment initiated.
- Worsening renal failure.
- Cardiac arrest and death of the patient.
- Culture with sensitive mycobacterium TB.

**Discussion**

- Diagnosis of TB often delayed or missed in the setting of ARDS.
- ARDS a rare complication of TB.
- Importance of early empiric treatment with antituberculous medications to decrease mortality.
- APACHE score >18 associated with higher mortality.
- Use of steroids controversial.
- High overall mortality described in TB and ARDS.
- High index of suspicion is important.
- Future studies needed to assess patients’ characteristics in TB and ARDS.
